# *Mycobacterium tuberculosis* Growth following Aerobic Expression of the DosR Regulon

**DOI:** 10.1371/journal.pone.0035935

**Published:** 2012-04-27

**Authors:** Kyle Minch, Tige Rustad, David R. Sherman

**Affiliations:** 1 Seattle Biomedical Research Institute, Seattle, Washington, United States of America; 2 Molecular and Cellular Biology Program, University of Washington, Seattle, Washington, United States of America; Fundació Institut d'Investigació en Ciències de la Salut Germans Trias i Pujol, Universitat Autònoma de Barcelona, CIBERES, Spain

## Abstract

The *Mycobacterium tuberculosis* regulator DosR is induced by multiple stimuli including hypoxia, nitric oxide and redox stress. Overlap of these stimuli with conditions thought to promote latency in infected patients fuels a model in which DosR regulon expression is correlated with bacteriostasis *in vitro* and a proxy for latency *in vivo*. Here, we find that inducing the DosR regulon to wildtype levels in aerobic, replicating *M. tuberculosis* does not alter bacterial growth kinetics. We conclude that DosR regulon expression alone is insufficient for bacterial latency, but rather is expressed during a range of growth states in a dynamic environment.

## Introduction

*Mycobacterium tuberculosis* is a remarkably successful pathogen that causes ∼9 million new cases of active tuberculosis (TB) disease every year [1]. However, this pool of patients with clinical disease is dwarfed by a vast reservoir of latently infected individuals estimated to consist of ∼1/3 of the world's population [2]. A prominent model of TB pathogenesis argues that a heterogeneous bacterial population within latently infected individuals enters into a reversible state of non-replicating persistence or dormancy, induced by diverse stimuli including nutrient deprivation, nitric oxide and hypoxia [3]. *In vitro* study of the latter condition has demonstrated that hypoxia can drive *M. tuberculosis* in to a viable but bacteriostatic state with concomitant substantial remodeling of the transcriptional and metabolic profile of the cell [4]. One of the earliest mediators of this transcriptional shift is induction of the two-component response regulator, Rv3133c [5].

Rv3133c was initially identified as a regulator differentially expressed in a virulent strain *M. tuberculosis* (DevR) [6]. Subsequently this transcription factor was shown to be induced in response to hypoxia [5], nitric oxide [7], or redox stress [8], and was renamed dormancy survival regulator (DosR) [9]. Induction of DosR results in the expression of ∼49 genes under its direct control. Furthermore, because each of these conditions is associated with aspects of bacterial dormancy *in vitro* and clinical latency, it was hypothesized that DosR induction initiated a genetic program that prepared *M. tuberculosis* for survival of bacteriostasis [7]. It appears, however, that DosR regulon induction during the initial hypoxic response is not absolutely required for survival during bacteriostasis (dormancy), as a *dosR* knock-out shows a modest survival defect upon exposure to short- or long-term defined hypoxic conditions [10]. In models of gradual oxygen depletion, genetic disruption of *dosR* or the three-gene *dosR* operon led to larger decreases in viability in *M. tuberculosis* and *M. bovis BCG* [9], [11], though it is unclear to what extent nutrient depletion or toxic metabolic by-products impact these observations. Despite uncertainty about the role of DosR in the natural history of TB disease, expression of DosR regulon genes is sometimes cited as evidence of impending *in vitro* dormancy, with implications for the management of clinical latency [12]. This association persists despite the observation that *M. tuberculosis* strains of the W-Beijing lineage constitutively express the DosR regulon [13].

In this work we directly address the question of *M. tuberculosis* growth rate following DosR regulon expression. We demonstrate that under aerobic conditions, ectopic induction of DosR is sufficient to induce the DosR regulon to near wild-type levels, even in the absence of its usual cues. However, this induction does not cause bacteriostasis or otherwise alter the growth kinetics of replicating *M. tuberculosis*.

## Materials and Methods

### Strains and Culturing Conditions

All experiments were performed using an Rv3133c/DosR knockout *M. tuberculosis* strain, H37Rv:Δ*dosR*, generated in our lab and described previously [14]. In the present work, H37Rv:Δ*dosR* was transformed with an episomal plasmid containing Rv3133c/DosR under the control of the smyc, anhydrotetracycline-inducible, promoter described by Ehrt, *et al* [15]. This vector contains a hygromycin B-resistance cassette and was modified to a contain Gateway Recombination™ (Invitrogen) cassette (kind gift of Eric Rubin). We further adapted this destination vector to contain an in-frame N-terminal FLAG epitope tag to create the vector pDTNF (plasmid Destination Tet. N-terminal Flag Tag). The *dosR* gene was transferred from the appropriate stock in an entry clone library (PFGRC, contracted by the NIAID) in to pDTNF to create the plasmid, pEXNF-3133c (EXpression N-terminal Flag Tag). Successful recombination was confirmed by sequencing (data not shown). The resulting ATc-inducible DosR complement strain, H37Rv:Δ*dosR*::*pEXNF-3133c*, was cultured in Middlebrook 7H9 with the ADC supplement (Difco),0.05% Tween80, and 50 µg/mL hygromycin B at 37°C with constant agitation. All experiments were performed under aerobic conditions and growth was monitored by OD600. Expression of pEXNF-3133c was induced using an ATc concentration of 10 ng/mL or 100 ng/mL culture. For uninduced controls, an equivalent volume of sterile DMSO was added to cultures. ATc-dependent expression of DosR was confirmed by α-DosR/α-FLAG western blot (data not shown) as well as by microarray transcriptional profiling.

### RNA Preparation

RNA was isolated as described previously [10]. Briefly, pellets in Trizol were transferred to a tube containing Lysing Matrix B (QBiogene, Inc.), and vigorously shaken at max speed for 30 sec in a FastPrep 120 homogenizer (Qbiogene) three times, with cooling on ice between steps. This mixture was centrifuged at max speed for 1 min and the supernatant was transferred to a tube containing 300 µL chloroform and Heavy Phase Lock Gel (Eppendorf North America, Inc.), inverted for two minutes, and centrifuged at max speed for five minutes. The aqueous phase was then precipitated with 300 µL isopropanol and 300 µL high salt solution (0.8 M Na citrate, 1.2 M NaCl). RNA was purified using an RNeasy kit following manufacturer's recommendations (Qiagen). Total RNA yield was quantified using a Nanodrop (Thermo Scientific).

### Microarray analysis

RNA was converted to Cy dye-labeled cDNA probes as described previously [10]. For all experiments described here, 1 µg of total RNA was used to generate probes. Sets of fluorescent probes were then hybridized to custom NimbleGen tiling arrays consisting of 135,000 probes spaced at ∼100 bp intervals around the *M. tuberculosis* H37Rv genome (NCBI Geo Accession #: GPL14896). Three biological replicate experiments of both induced and uninduced cultures were hybridized to arrays. Arrays were scanned and spots were quantified using Genepix 4000B scanner with GenePix 6.0 software. These data were exported to NimbleScan for mask alignment, and ArrayStar for robust multichip average (RMA)[16] normalization and statistical analysis (NCBI GEO Accession #: GSE33752). Altered gene expression was considered significant if it produced a moderated t-test P<0.05 after Benjamini Hochberg multiple testing correction.

## Results

### Aerobic ectopic induction of DosR results in expression of the DosR regulon

**Figure 1:**
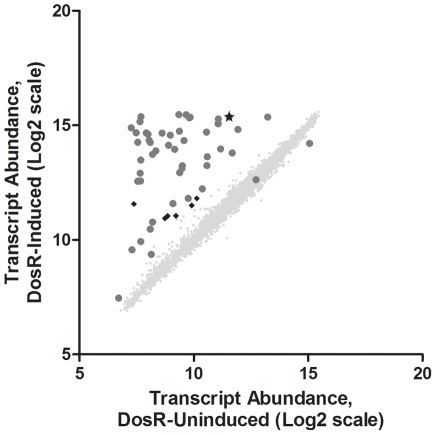
Ectopic expression of DosR induces the DosR regulon. Scatterplot displaying transcript levels of all *M. tuberculosis* genes after 24 hours of treatment with either 10 ng/mL Atc (induced) or an equivalent volume of sterile DMSO (uninduced). Three biological replicates were RMA-normalized and the median pixel intensity data are plotted on a log_2_ scale. Genes of the DosR regulon are represented as dark gray circles. Significantly induced genes (moderated t-test with Benjamini-Hochberg FDR correction, p<0.05) not part of the DosR regulon are presented as black diamonds, and the *dosR* transcript is indicated with a star.

The DosR regulon was originally described as those *M. tuberculosis* genes induced following 2 hours of hypoxic gas treatment (0.2% O_2_) [5], a condition thought to mimic aspects of the granuloma during latent infection that results in bacteriostasis *in vitro*. Because expression of the DosR regulon represents the first broad transcriptional adaptation to hypoxia, it has been thought to play a critical role in driving a survival adaptation during this stress condition. To determine the growth-rate implications of aerobic DosR expression, we used a *ΔdosR M. tuberculosis* genetic background previously generated in our lab [14], and created a complement strain in which DosR could be conditionally expressed with the addition of anhydrotetracycline (ATc). We first established if the addition of ATc was sufficient to induce the DosR regulon in rolling culture. Early log phase cultures were diluted to OD600 of 0.04 and incubated independently for 24 hours prior to chemical induction of DosR. At T0 cultures were treated with 10 ng ATc (dissolved in DMSO) per mL of culture, or a volume-matched amount of sterile DMSO, and RNA was collected at 12 and 24 hours post-induction. Three biological replicates of induced and three uninduced samples were processed and hybridized to genome-wide tiling microarrays. Focused transcriptional changes were apparent at 12 hours (data not shown), and by 24 hours of ectopic DosR induction under aerobic conditions, 50 genes were significantly induced, including 44 genes of the traditionally-defined DosR regulon (Figure 1). Interestingly, these genes were induced under conditions in which neither of the histidine kinases known to interact with DosR have documented activity – log phase aerobic growth. Two DosR regulon genes, Rv3126c (conserved hypothetical) and Rv3132c (DosS) approached but did not reach statistical criteria for significant induction. Rv3841, a gene noted previously to be mildly induced during early hypoxia and considered part of the DosR regulon but without a canonical DosR binding motif [14], was moderately repressed under these conditions, raising the possibility this gene is not a member of the DosR regulon, but rather is correlatively induced in hypoxia by another factor not under the control of DosR. Along with ∼90% of the DosR regulon, 6 additional genes (Rv0085, Rv0086, Rv0087, Rv1519, Rv1735c, and Rv2386c) were found to be significantly upregulated in response to this treatment. With the exceptions of Rv1519 and Rv2386c, all of these genes are located in operons with, or adjacent to, genes of the DosR regulon with known DosR binding sites.

To examine the ectopic response more closely we next asked whether the aerobic induction of DosR and the DosR regulon produced an overall transcriptional landscape similar to that of bacilli after 2 hours of hypoxic gas treatment. To do this, we performed a meta-analysis comparing the aerobic/ectopically induced DosR regulon expression profile to the earlier reported DosR transcriptional data [14]. We found a significant correlation between the expression levels observed under ectopic/aerobic conditions and native DosR/DosR regulon expression under hypoxic conditions (Spearman r = 0.672, P<0.0001). We thus conclude that ectopic/chemical induction of Rv3133c from the ATc-inducible plasmid pEXNF-3133c under aerobic conditions results in expression of the DosR regulon comparable to that of the initial hypoxic response.

### Ectopic induction of the DosR regulon does not alter growth kinetics of *M. tuberculosis*

**Figure 2:**
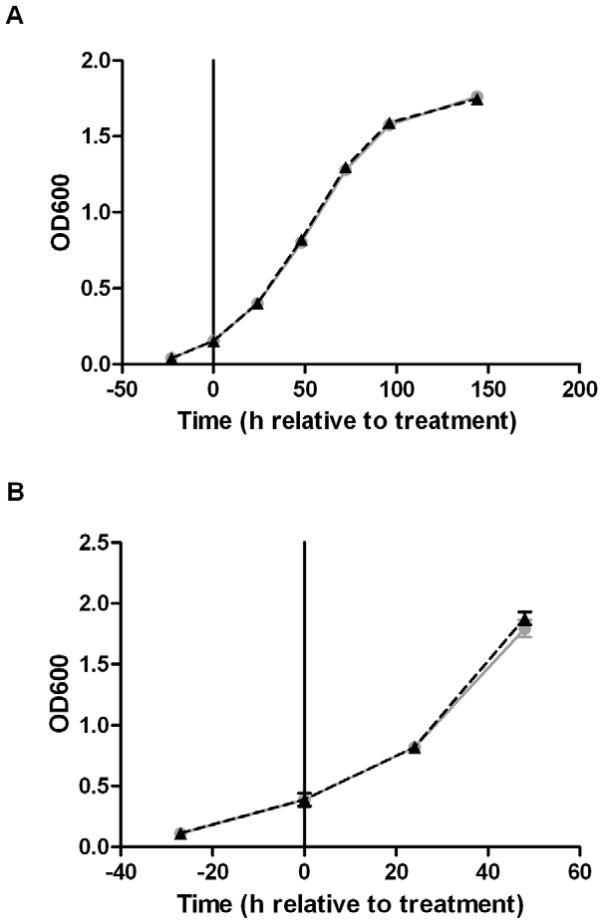
DosR regulon expression does not alter *M. tuberculosis* growth kinetics. A) Growth curves of cultures in which DosR was either ectopically induced with 10 ng/mL ATc (gray circles connected by solid line) or treated with an equivalent volume sterile DMSO (uninduced, black triangles connected by dashed line). OD600 of three biological replicates were tracked for 6 days following chemical treatment. Displayed are mean OD600 +/− standard deviation. Doubling times for induced (21.97 hours) and uninduced (21.54 hours) cultures were calculated using exponential growth equation from nonlinear regression fit of un-/induced OD600 data points. Time points included in doubling time calculation were T0, T24, and T48. B) Growth curves of *M. tuberculosis* in which DosR was either ectopically induced with 100 ng/mL ATc or treated with an equivalent volume of sterile DMSO. OD600 of three biological replicates were tracked for 48 hours following chemical treatment. Strain identifiers as described in 2a. Doubling times for induced (21.19 hours) and uninduced (20.33 hours) cultures calculated as above.

Having established conditions in which DosR regulon induction is uncoupled from its traditional signals, we next sought to determine the impact on the growth kinetics of replicating *M. tuberculosis*. Using the induction conditions described for transcriptional profiling above, Figure 2A shows a growth curve in which expression of the DosR regulon was induced in early log phase (T0). Over the entire time course it is apparent that DosR regulon expression had no impact on the doubling time or entrance into stationary phase when compared with the uninduced control. To investigate if replication rate was more sensitive to DosR regulon induction at different growth phases, we also assessed the impact of chemical DosR induction during mid-log phase. Using bacteria at OD600 of 0.4 and ten times more ATc (100 ng/mL) than before, growth rate was again unaffected (Figure 2B). We conclude that induction of the DosR regulon does not by itself establish a bacteriostatic phenotype, or alter *M. tuberculosis* growth kinetics.

## Discussion

In this work we investigated the impact of DosR regulon expression on replicating *M. tuberculosis* under aerobic conditions. Utilizing an *M. tuberculosis* H37Rv *dosR* knockout strain complemented with an ATc-inducible copy of *dosR*, we found that ectopic expression of DosR under aerobic conditions resulted in a transcriptional pattern strikingly similar to that found when wild-type *M. tuberculosis* is exposed to 0.2% oxygen for 2 hours – a condition that results in bacterial growth arrest. However, expression of the DosR regulon to these near-physiological levels had no effect on growth rates after induction compared to DosR-uninduced cultures. Over a period of several days, these cultures replicated with nearly identical kinetics and displayed no differences as they entered stationary phase. Previous studies indicate that disruption of *dosR* can produce a range of hypoxic survival phenotypes, with survival defects of ∼1.5 log to ∼4 log reported under long-term hypoxic conditions [9], [10], [11]. However, we have demonstrated that deletion of *dosR* had little impact on the long-term transcriptional adaptation of *M. tuberculosis* to hypoxic environments [10]. Thus, the precise role of DosR regulon induction remains an open question, but the data reported here lead us to conclude that it is not sufficient to initiate bacteriostasis.

Results reported here differ from a study performed using *M. bovis BCG* in which a constitutively-expressed merodiploid copy of DosR induced fewer members of the DosR regulon, and resulted in a moderate growth defect [17]. Perhaps the difference in species used accounts for this discrepancy. In addition, we cannot exclude the possibility that DosR-dependent growth arrest requires precise induction of a particular “native” transcriptional profile not reproduced in the ectopic system described here. However, as noted above, the transcriptome generated by ectopic DosR expression is remarkably similar to that produced after 2 hours of hypoxia, and those few genes demonstrating the greatest expression differences in this system do not have functions clearly associated with growth arrest.

The observation that *M. tuberculosis* replication is not affected by expression of the DosR regulon does not preclude the possibility that genes from this regulon are expressed during periods of clinically latent disease. This scenario is consistent with recent hypotheses that the term “latent infection” is a broad classifier describing a number of different states [4], [18]; however, that these genes are expressed during active replication strongly argues that DosR regulon expression is not an explicit marker of latent infection. Indeed, it appears that expression of the DosR regulon correlates with multiple stages of infection and disease in a dynamic host environment.
